# Post-radiation sarcoma: A study by the Eastern Asian Musculoskeletal Oncology Group

**DOI:** 10.1371/journal.pone.0204927

**Published:** 2018-10-17

**Authors:** Min Wook Joo, Yong Koo Kang, Koichi Ogura, Shintaro Iwata, June Hyuk Kim, Won Ju Jeong, Xiaohui Niu, Pramod S. Chinder, Han Soo Kim, Sung Wook Seo, Yang-Guk Chung

**Affiliations:** 1 Department of Orthopaedic Surgery, St. Vincent's Hospital, College of Medicine, The Catholic University of Korea, Banpo 4-dong, Seocho-gu, Seoul, Republic of Korea; 2 Department of Musculoskeletal Oncology, National Cancer Center Hospital, Tsukiji, Chuo-ku, Tokyo, Japan; 3 Orthopaedic Oncology Service, National Cancer Center, Goyang, Republic of Korea; 4 Daegu Top Hospital, Daegu, Republic of Korea; 5 Department of Orthopedic Oncology Surgery, Beijing Ji Shui Tan Hospital, Beijing, Xicheng District, China; 6 Department of Musculoskeletal Oncology, HCG Bangalore Institute of Oncology, Bangalore, India; 7 Department of Orthopaedic Surgery, Seoul National University Hospital, aehak-ro, Jongno-gu, Seoul, Republic of Korea; 8 Department of Orthopedic Surgery, Samsung Medical Center, Sungkyunkwan University School of Medicine, Irwon-ro, Gangnam-Gu, Seoul, Republic of Korea; 9 Department of Orthopaedic Surgery, Seoul St. Mary’s Hospital, College of Medicine, The Catholic University of Korea, Seocho-gu, Seoul, Republic of Korea; Kanazawa University, JAPAN

## Abstract

The oncologic risk of ionizing radiation is widely known. Sarcomas developing after radiotherapy have been reported, and they are a growing problem because rapid advancements in cancer management and screening have increased the number of long-term survivors. Although many patients have undergone radiation treatment in Asian countries, scarce reports on post-radiation sarcomas (PRSs) have been published. We investigated the feature and prognostic factors of PRSs in an Asian population. The Eastern Asian Musculoskeletal Oncology Group participated in this project. Cases obtained from 10 centers were retrospectively reviewed. Patients with genetic malignancy predisposition syndrome, or who had more than one type of malignancy before the development of secondary sarcoma were excluded. Forty-two high-grade sarcomas among a total of 43 PRSs were analyzed. There were 29 females and 13 males, with a median age of 58.5 years; 23 patients had bone tumors and 19 had soft tissue tumors. The most common primary lesion was breast cancer. The median latency period was 192 months. There were no differences in radiation dose, latency time, and survival rates between bone and soft tissue PRSs. The most common site and diagnosis were the pelvic area and osteosarcoma and malignant fibrous histiocytoma for bone and soft tissue PRSs. The median follow-up period was 25.5 months. Five-year metastasis-free and overall survival rates were 14.5% and 16.6%, and 39.1% and 49.6% for bone and soft tissue PRSs. Survival differences depending on initial metastasis and surgery were significant in soft tissue sarcomas. Although this study failed to find ethnic differences, it is the largest review on PRS in an Asian population. As early recognition through long-term surveillance is a key to optimal management, clinicians should take efforts to understand the real status of PRS.

## Introduction

The oncologic risk of ionizing radiation is widely known [1]. In 1922, bone sarcomas developing after radiotherapy in patients with benign conditions were reported as one of the first solid malignant tumors related to radiotherapy, which implicate radiation treatment as one of the pathogeneses of sarcomas [2]. In 1948, 11 cases of post-radiation osteosarcoma (PRS) were first reported in the English literature [3]. A recent analysis of the Surveillance, Epidemiology, and End Results (SEER) registries demonstrated that the risk of second primary bone sarcoma in patients who had received radiotherapy was increased by 257% compared to that of primary bone sarcoma in the general population [4]. PRSs are uncommon, and they account for 0.5–5% of all sarcomas [1, 5]. While the precise incidence is not known, one of the most comprehensive studies demonstrated that the cumulative incidence was estimated to be less than 1% at 15 years after the previous cancer diagnosis [6]. However, PRSs are a growing problem and a critical clinical subgroup of sarcomas because rapid advancements in cancer management and screening have increased the number of long-term cancer survivors [1, 7].

The number of cancer patients has been increasing rapidly in Asian countries. Lifestyle changes, urbanization, changes in reproductive patterns, westernized diet and obesity, and increasing lifespans have contributed to the growing incidence of cancer [8]. As radiotherapy reduces cancer mortality and recurrence [4], it has become one of the most important modalities in the management of various solid cancers [1]. In Japan, 726 facilities delivered radiation therapy for 149793 new patients in 2003 [9]. The Study on the Current Status of Radiotherapy in Korea has reported that the number of patients who underwent radiation treatment increased by 65% from 37215 among 154552 cancer patients in 2006 to 56850 among 224177 cancer patients in 2013 [10]. In spite of such a situation, scarce reports on PRSs in an Asian population have been published to date. We performed a retrospective multi-national, multi-institutional study to investigate the clinicopathological feature, and prognostic factors of PRSs in an Asian population.

## Materials and methods

Cases obtained from 10 tertiary musculoskeletal oncology centers in four nations were retrospectively reviewed. Based on the existing diagnostic criteria for PRS [3, 11, 12], we included the following subjects: (1) patients with histologically proven sarcoma which developed within the external beam radiotherapy field; (2) a latency period of more than three years between radiation therapy and appearance of sarcoma. Patients with genetic malignancy predisposition syndrome such as familial retinoblastoma, Li-Fraumeni syndrome, or Rothmund-Thomson syndrome, who had secondary sarcomas at sites distant to the radiation field, who received brachytherapy or total-body irradiation, or who had more than one type of malignancy before the development of secondary sarcoma were excluded from this study. This study was approved by the Institutional Review Board of Catholic Medical Center in Korea. Because we conducted a retrospective review and this was a minimal risk study, and we did not collect any personally identifiable information, any informed consents were not collected from participants.

Data on demographics, primary lesion, and secondary sarcoma was collected. With respect to the primary lesion, the location, histological diagnosis and subtype, radiation therapy, and chemotherapy were evaluated. With respect to secondary sarcoma, histological diagnosis including its subtype and grade, main length and volume, the latency period from radiotherapy, surgery and surgical margin, local recurrence, distant metastasis, oncologic outcome, and follow-up period were assessed. Tumor volume was measured as π/6 x (length) x (width) x (height). Follow-up period was defined as the interval from the diagnosis of PRS to death or the last visit.

Latency period was defined as the interval from the start date of radiotherapy for the primary lesion to the date of recognition of secondary sarcoma development. Distinction in the radiation dose and latency period between bone and soft tissue lesions was estimated by normality test and Student’s t-test. The differences in metastasis-free survival rate (MFSR) and overall survival rate (OSR) depending on bone and soft tissue sarcomas were estimated using the log-rank test by univariate analysis. OSR and MFSR were defined as the periods from the date of diagnosis of secondary sarcoma to the date of death or last follow-up, and to the date of recognition of distant metastasis. These rates were calculated using Kaplan-Meier survival curves. The influence of potential prognostic factors was evaluated using the log-rank test by univariate analysis. A p-value below 0.05 was considered statistically significant. SPSS 21.0 for Windows (SPSS Corporation, Chicago, IL) was used for statistical analysis.

## Results

### Patients

Details of the 43 cases are presented in Table 1. Except for one patient with a low-grade tumor (the 10th soft tissue sarcoma case in Table 1), a total of 42 patients with high-grade tumors were analyzed. There were 29 females and 13 males, with a median age of 58.5 years (range, 13–80 years) at the initial diagnosis of high-grade PRS; 23 patients had bone tumors and 19 had soft tissue tumors. All patients underwent external beam radiotherapy for a benign, borderline, or malignant lesion; the most common histological diagnosis of the primary lesion was breast cancer. The median radiation dosage for the primary lesion was 49.7 Gy (range, 12–72 Gy) in 20 patients for whom data was available, and most of them, except for one patient with the lymphoma of bone, received a minimum dose of 30 Gy. Fourteen patients had undergone chemotherapy for their primary tumor with various combinations of chemotherapeutic agents; alkylating anti-neoplastic agents were administered to four of the five patients for whom related details were available. In the other eight patients, it could not be identified whether chemotherapy was administered. The median latency period was 192 months (range, 46–503 months) in 37 patients for whom data was available. There were no significant differences in radiation dose and latency time according to bone or soft tissue sarcoma in patients for whom data was available (p = 0.235, and 0.922). Thirty-three patients had undergone surgery for PRS; surgical margins were wide in 28 patients, marginal in two patients, and intra-lesional in three patients. Among the nine patients who did not undergo surgery, the median age was 74 years (range, 25–80 years), the median main length and volume of the lesion was 8.6 cm (range, 6-17cm) and 151.16 cm^3^ (range, 25.10–1176.75 cm^3^), respectively, and eight patients had bone sarcomas. Among them, only one patient had initial metastasis, and seven patients had lesions in the pelvis and two patients had lesions in the skull. None of the patients had again received radiotherapy for PRS.

**Table 1:** Patient details.

	Primary lesion			Post-radiation sarcoma					
Patient No./Gender/Age (years)	Histology	RTx dose (Gy)/Frx	CTx	Histology/Subtype	Latency period (months)	Location	Size (cm^3^)	Initial metastasis	Surgery/Margin
Bone sarcoma									
1/F/46	Plasmacytoma	N/A	No	OSA	187	Pelvis	7 x 7 x 8.5	-	No
2/M/25	Retinoblastoma	49.4/26	No	OSA	287	Temporal bone	10 x 6 x 1	-	No
3/F/78	Cancer of the buccal mucosa	30	TGF, CBDCA	OSA	281	Mandible	4 x 3.7 x 3.5	-	Resection/W
4/F/74	Cervical cancer	70	N/A	Leiomyosarcoma of bone	108	Pelvis	6.4 x 7.9 x 8.6	-	No
5/F/64	Cervical cancer	N/A	N/A	OSA	276	Pelvis	5.6 x 4 x 7.1	+	LSS/W
6/M/45	Langerhans cell histhiocytosis	N/A	No	OSA	360	Tibia	4.5 x 4.3 x 6.5	-	LSS/W
7/F/60	Rectal cancer	N/A	Yes	UPS of bone	204	Femur	2 x 3 x 3	-	LSS/W
8/M/60	Prostate cancer	N/A	No	OSA/Osteoblastic	N/A	Pelvis	5 x 10 x 20	-	LSS/W
9/F/57	Uterine cancer	50	Yes	OSA/Conventional	N/A	Pelvis	10 x 15 x 15	-	No
10/F/46	Uterine cancer	N/A	Yes	OSA/Osteoblastic	N/A	Pelvis	6 x 7 x 17	-	No
11/M/75	Prostate cancer	N/A	No	OSA/Chondroblastic	N/A	Pelvis	4 x 8.5 x 8.5	-	No
12/F/63	Uterine cancer	N/A	No	OSA/Osteoblastic	N/A	Pelvis	10 x 7 x 22	-	LSS/W
13/F/53	Cervical cancer	N/A	No	OSA/Fibroblastic	228	Coccyx	6 x 8 x 11	-	LSS/W
14/F/40	GCT of bone	N/A	No	OSA/Fibroblastic	180	Tibia	6 x 7 x 8	+	LSS/W
15/F/72	Breast cancer	50/25	No	Chondrosarcoma	192	Humerus	4 x 6 x 6	+	Amputation/W
16/F/47	Breast cancer	N/A	N/A	OSA/Chondroblastic	228	Sterum	4 x 5 x 6	-	Resection/W
17/F/76	Cervical cancer	N/A	N/A	UPS of bone	96	Pelvis	2 x 3 x 16	-	No
18/F/63	Cervical cancer	N/A	N/A	OSA/Osteoblastic	148	Pelvis	9 x 12 x 16	-	Resection/M
19/F/34	Glioma	N/A	N/A	OSA/Osteoblastic	264	Skull	2 x 4 x 6	-	No
20/F/70	Breast cancer	N/A	N/A	UPS of bone	408	Sternum	3 x 4 x 4	-	Resection/W
21/F/57	Breast cancer	N/A	N/A	OSA/Chondroblastic	156	Rib	13.1 x 13.2 x 14.8	-	Resection/W
22M/74	Squamous cell carcinoma	66	No	OSA/Fibroblastic	227	Mandible	1 x 1.1 x 1.3	-	Resection/W
23/M/43	Liposarcoma	N/A	No	OSA/Osteoblastic	156	Femur	2 x 2.3 x 3.7	+	Resection/W
Soft tissue sarcoma									
1/F/27	Retinoblastoma	40/20	No	RMS/Embryonal	319	Ethmoid sinus	4.5 x 2.5 x 4	-	Resection/W
2/M/47	Hypopharangeal cancer	72	CDDP, 5-FU	UPS	145	Neck	6 x 6 x 4	-	Resection/W
3/F/68	Uterine cancer	50.4	No	Angiosarcoma	121	Abdominal wall	5 x 3.5 x 1	-	Resection/W
4/F/53	Uterine cervical cancer	N/A	No	Fibrosarcoma	144	Buttock	14.9 x 12.8 x 7	-	Resection/M
5/F/72	Breast cancer	50	Yes	Fibrosarcoma	46	Chest wall	7 x 6 x 3	-	Resection/W
6/M/32	Hemangioendothelioma	N/A	No	Fibrosarcoma	383	Chest wall	5.7 x 4.5 x 5	-	ResectionW
7/F/65	Uterine cancer	50	No	Angiosarcoma	75	Abdominal wall	3 x 3 x 1	-	Resection/I
8/M/32	Retinoblastoma	40/20	No	UPS	370	Ethomoid sinus	1 x 1 x 1	-	Resection/W
9/M/73	Tongue carcinoma	40.8/17	No	UPS	503	Chin	4 x 3 x 2.2	-	Resection/W
10/F/75	Breast cancer	50/25	No	Fibrosarcoma	443	Chest wall	8.7 x 3.7 x 7	-	No
11/F/42	Lymphoma of bone	12/6	ADM, BHAC, 6-MP	UPS	321	Head	6.5 x 4.4 x 5.5	-	Resection/W
12/M/25	Retinoblastoma	46/23	CDDP, Thiotepa	RMS	295	Orbital cavity	3.6 x 2.2 x 3.5	-	Resection/I
13/F/63	Uterine cervical cancer	30	Yes	UPS	177	Abdominal wall	11 x 9 x 7.5	-	Resection/W
14/M/13	Retinoblastoma	40	Yes	UPS	160	Accessory sinus	4 x 3.2 x 1.8	-	Resection/W
15/M/70	Esophageal cancer	30	Yes	Myxofibrosarcoma	167	Back	8 x 5 x 8	-	Resection/I
16/F/61	Breast cancer	50/25	Epirubicin, CDDP, 5-FU	Synovial sarcoma	50	Shoulder	6 x 8 x 6	+	Amputation/W
17/F/61	Breast cancer	N/A	No	Sarcoma, NOS	228	Neck	2 x 4 x 5	-	Resection/W
18/F/80	Cervical cancer	N/A	No-	Sarcoma, NOS	444	Pelvis	5 x 7 x 8	+	No
19/F/43	Rectal cancer	63	Yes	UPS	57	Buttock	1.3 x 2.1 x 2.5	-	Resection/W
20/F/56	Breast cancer	N/A	Yes	OSA/Extraskeletal	N/A	Shoulder	6.5 x 7.5 x 10.5	-	LSS/W

GCT Giant cell tumor, RTx Radiotherapy, Frx Fraction, N/A Not applicable, TGF Transforming growth factor, CBDCA carboplatin, CDDP Cisplatin, 5-FU 5-fluorouracil, ADM Adriamycin, BHAC, Behenoyl cytosine arabinoside, 6-MP 6-mercaptopurine, OSA Osteosarcoma, UPS Undifferentiated pleomorphic sarcoma, RMS Rhabdomyosarcoma, NOS Not otherwise specified, W Wide, LSS Limb-sparing surgery, M Marginal, I Intralesional

**Fig 1:**
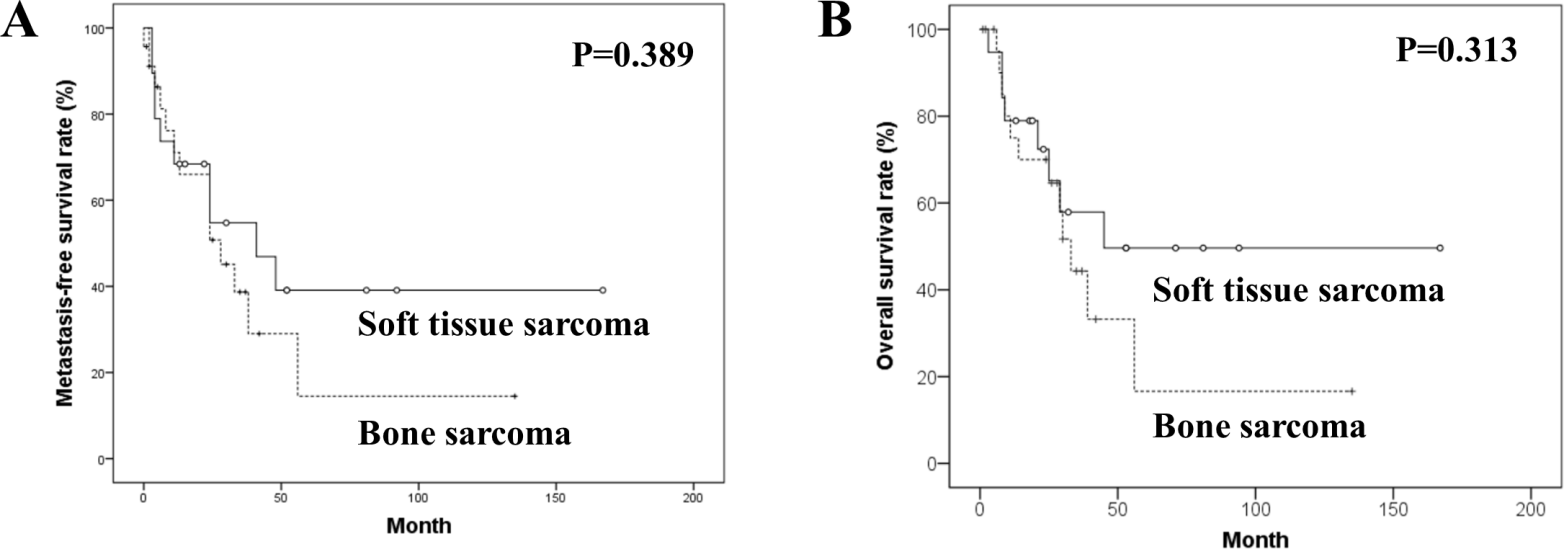
Kaplan-Meier survival curves. Kaplan-Meier curves for (A) metastasis-free and (B) overall survivals in the bone and soft tissue post-radiation sarcomas.

The median follow-up periods were 25.5 months (range, 1 to 167 months) in all patients and 33.5 months (range, 5 to 167 months) in 18 survivors. The 5-year MFSR and OSR were 26.3% and 34.4%. The differences in both these survival rates between bone and soft tissue sarcomas were not significant (p = 0.389, and 0.313) (Fig 1).

### Bone sarcomas

PRSs of the bone were observed in 17 females and six males with a median age of 60 years (range, 25–78 years). The median main length and volume of the tumors were 8.5 cm (range, 1.3–22 cm) and 117.17 cm^3^ (range, 0.75–1338.47 cm^3^), respectively. Common primary lesions were cancer of the female genital system and breast carcinoma. The most common location and pathologic diagnosis were the pelvis (10) and osteosarcoma (18). All bone sarcomas were high-grade. Four patients had undergone chemotherapy for the primary lesion. In eight patients, it could not be identified whether chemotherapy was administered. The median latency period was 215.5 months (range, 96–408 months) in 18 cases for whom data was available. Fifteen patients underwent surgical treatment for their sarcoma; surgical margins were wide in 14 patients and marginal in one patient. Among eight patients who did not undergo surgery, the median age was 51.5 years (range, 25–76 years), and none of them had distant metastasis at presentation. Among them, six patients had lesions in the pelvis and two patients had lesions in the skull.

**Fig 2:**
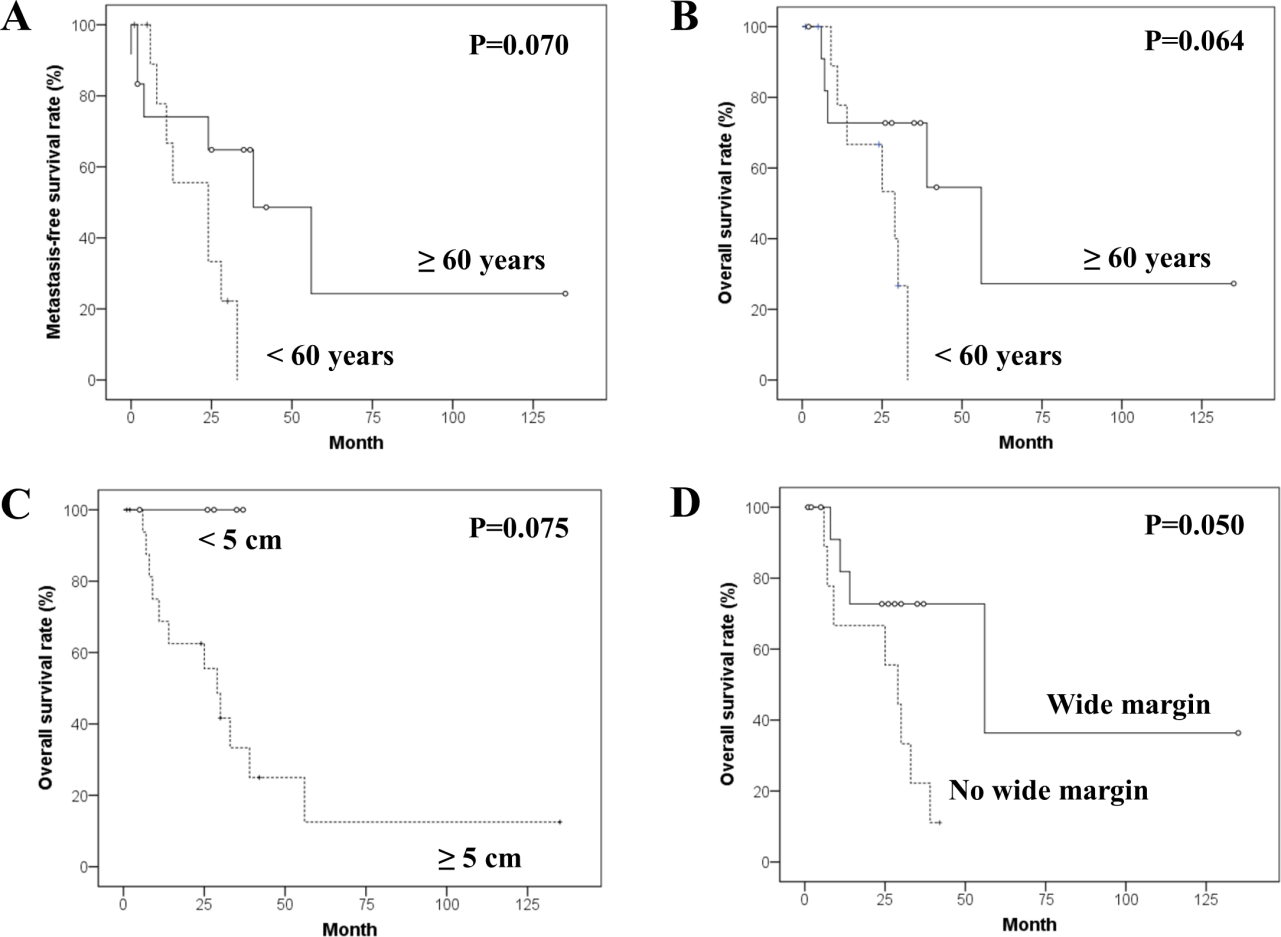
Kaplan-Meier survival curves in univariate analyses for the bone post-radiation sarcoma. Kaplan-Meier curves for (A) metastasis-free survival according to age, and for overall survivals according to (B) age, (C) main length and (D) surgical margin.

The median follow-up periods were 26 months (range, 1 to 135 months) in all 23 patients and 35 months (range, 5 to 135 months) in 11 survivors. The 5-year MFSR and OSR were 14.5% and 16.6%. Prognostic factor analyses of MFSR and OSR are summarized in Table 2. The difference in MFSR according to age (≥60 years vs <60 years) approached statistical significance (p = 0.070). The differences in OSR depending on age (≥60 years versus <60 years), main length (≥5 cm versus <5 cm), and achieving a wide resection (yes versus no) were also insignificant; however, they showed interesting trends (Fig 2).

**Table 2:** Prognostic factor analyses of OSR and MFSR in patients with post-radiation bone sarcoma.

Factors		Univariate analysis				
		*N* (%)	5-year OSR %	*p* value	5-year MFSR %	*p* value
Age	< 60 years	11 (47.8)	0	0.064	0	0.070
	≥ 60 years	12 (52.2)	27.3		24.3	
Gender	Male	6 (26.1)	37.5	0.188	30.0	0.240
	Female	17 (73.9)	0		0	
Length	< 5 cm	5 (21.7)		0.075		0.142
	≥ 5 cm	18 (78.3)	12.5		11.4	
Initial metastasis	Yes	4 (17.4)	0	0.980	0	0.690
	No	19 (82.6)	28.0		25.8	
Surgical treatment	Yes	15 (69.6)	33.3	0.151	25.8	0.324
	No	8 (30.4)				
Wide margin	Yes	14 (60.9)	36.4	0.050	27.9	0.132
	No	9 (39.1)				

OSR overall survival rate, MFSR metastasis-free survival rate

### Soft tissue sarcomas

High-grade PRSs of the soft tissue were identified in 12 females and seven males with a median age of 56 years (range, 13–80 years). The median main length and volume of the tumors were 5.7 cm (range, 1–14.9 cm) and 65.90 cm^3^ (range, 1–698.23 cm^3^), respectively. The most common primary lesion was carcinoma of the female genital system. The most common histologic diagnosis was undifferentiated pleomorphic sarcoma (6). The median radiotherapy dose for the primary tumor was 43.4 Gy (range, 12–72 Gy) in 14 patients for whom data was available. Ten patients were treated by chemotherapy for their primary lesion. The median latency period was 167 months (range, 46–503 months). Eighteen patients had undergone surgery for PRS; surgical margins were wide in 14 patients, marginal in one patient, and intralesional in three patients. An 80-year-old female with distant metastasis at presentation did not undergo surgical resection of pelvic sarcoma with a main length of 8 cm and a volume of 146.44 cm^3^.

**Fig 3:**
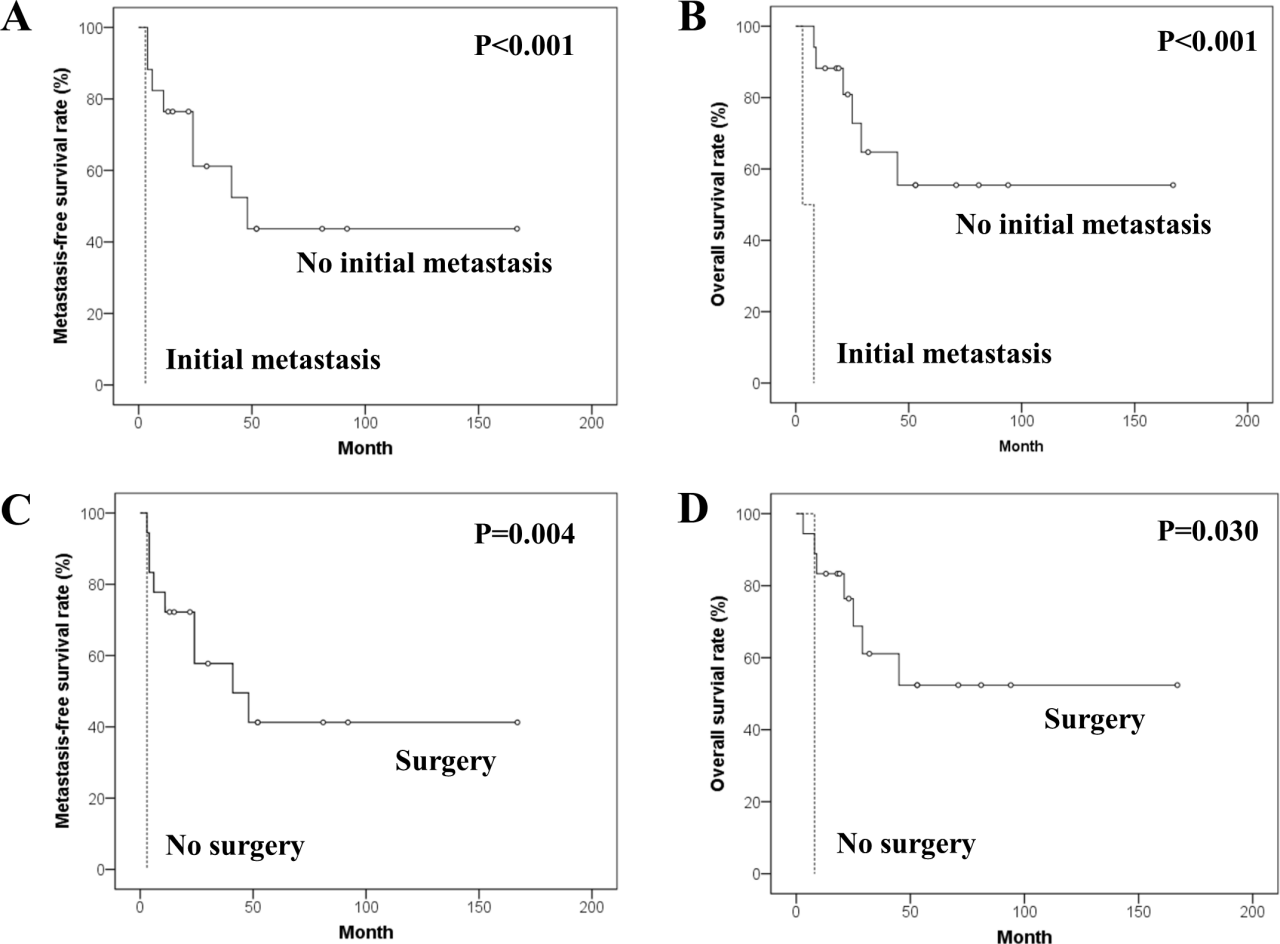
Kaplan-Meier survival curves in univariate analyses for the soft tissue post-radiation sarcoma. Kaplan-Meier curves for (A) metastasis-free and (B) overall survivals according to initial metastasis, and for (C) metastasis-free and (D) overall survivals according to surgical treatment.

The median follow-up periods were 25 months (range, 3–167 months) in all patients and 53 months (range, 13–167 months) in 9 survivors. Local recurrence developed in 5 patients with wide surgical margin. The 5-year MFSR and OSR were 39.1% and 49.6%. Prognostic factor analyses for MFSR and OSR are summarized in Table 3. The differences in MFSR and OSR depending on initial metastasis (yes versus no) were statistically significant (p<0.001, respectively). The differences in MFSR and OSR according to the surgical treatment (yes versus no) were also significant (p = 0.004 and p = 0.030) (Fig 3).

**Table 3:** Prognostic factor analyses of OSR and MFSR in patients with post-radiation soft-tissue sarcoma.

Factors		Univariate analysis				
		*N* (%)	5-year OSR %	*p* value	5-year MFSR %	*p* value
Age	< 60 years	10 (52.6)	57.1	0.154	38.6	0.343
	≥ 60 years	9 (47.4)	44.4		44.4	
Gender	Male	7 (36.8)	53.3	0.293		0.849
	Female	12 (63.2)	44.4		46.7	
Length	< 5 cm	7 (36.8)	57.1	0.469		0.614
	≥ 5 cm	12 (63.2)	46.9		41.7	
Initial metastasis	Yes	2 (10.5)	0	<0.001*	0	<0.001*
	No	17 (89.5)	55.5		43.7	
Surgical treatment	Yes	18 (94.7)	52.4	0.030*	41.3	0.004*
	No	1 (5.3)	0		0	
Wide margin	Yes	14 (73.7)	54.4	0.545	40.8	0.685
	No	5 (26.3)	30.0		30.0	
Age at diagnosis of primary lesion	< 20 years	6 (31.6)	60.0	0.343	40.0	0.410
	≥ 20 years	13 (68.4)	47.5		43.1	
Previous chemotherapy	Yes	10 (52.6)	61.7	0.164	54.9	0.151
	No	9 (47.4)	35.6		18.5	
Latency period	< 15 years	11 (57.9)	60.6	0.669	53.0	0.563
	≥ 15 years	8 (42.1)	36.5		18.8	

OSR overall survival rate, MFSR metastasis-free survival rate

*Statistically significant

## Discussion

We conducted this study to understand the current status of PRS in an Asian population and to explore the differences from the existing results of Western researches. Although recent rapid advances in tumor treatment and surveillance have improved the prognosis, comprehension of treatment-related risks seems to be too inadequate to reduce the therapy-related long-term complications, especially in Asia.

A multi-national, multi-institutional retrospective study inevitably has a potential for selection and management bias. Detailed information of some cases was not available. However, given that these diseases are rare and have been reported rarely in an Asian population, these results may be useful. As we have no data on the patients who had received radiation therapy within the same period as this study population, even an approximate risk of PRSs, potentially one of the most important points in an Asian study, could not be estimated.

The age of patients with PRS is determined by the age at radiotherapy for the first cancer and subsequent latency period, and it has been reported to range from five to 86 years [5, 13–15]. Cohort and registry researches have reported that medical radiation treatment increases the risk of subsequent bone sarcoma from approximately two times in adults to more than 100 times in some pediatric populations [16]. While previous studies have suggested a difference in susceptibility to radiotherapy between children and adults and an inverse relationship between age at radiation exposure and risk of osteosarcoma development [17, 18], more recent reports have proposed that the risk may increase again in the elderly, which potentially implies that radiation acts as a cancer promoter rather than a cancer initiator [19, 20]. However in this study, the details of children and adult patients were not analyzed separately. Age at radiotherapy might also affect the latency period [12, 21–23]. Previous literatures have reported that mean latency periods for post-radiation soft tissue sarcoma and bone sarcoma range from 6 to 18 years [21, 24–28] and from 4 to 17 years [11, 21, 22, 28–33], respectively. However, they also showed a wide range of latency periods [34], and a case developing after 65 years post-radiation has been reported [5]. While it is still controversial whether the latency periods for post-radiation soft tissue sarcoma and bone sarcoma are significantly different, some studies have demonstrated that the period may be longer in bone sarcoma [12], and this study did not show a statistical difference. Several reports have shown that the latency period seemed to be inversely associated with the radiation dose [14, 35]; however, the others reports presented contrary data [21, 26, 36, 37]. A study [4] showed that the latency period in radiation-associated chondrosarcoma was longer than that in osteosarcoma, and it suggested that this may be because chondrosarcoma typically shows slower growth with less metastasis than osteosarcoma. Additional enlightenment on the expected latency period for individual conditions may lead to the development of an efficient strategy for patient education and surveillance and may result in an expeditious differential diagnosis [5, 15].

Previous researches have shown that common primary diagnoses of PRSs were soft tissue neoplasms, such as breast cancer [3, 11–15, 26, 29–33, 37–42], lymphoma [12–14, 21, 26, 29, 30, 32, 39, 40, 43], genitourinary cancer [13, 14, 21, 26, 27, 29–33, 38, 40], and head and neck cancer [5]. Particularly with respect to breast carcinoma, the first case arising in the chest wall after radiation was reported in 1976 [44]. This study distinctively included five cases with retinoblastoma. A recent study has revealed a difference in standardized incidence ratios for subsequent PRS according to the type of first cancer [45].

As PRS is often locally aggressive, and therefore, it has already advanced and not resectable or may metastasize at initial diagnosis, it is related to a poor overall prognosis [5, 7, 15, 28, 46]. Local recurrence rates ranged from 17% to 68% [15, 28, 47, 48], and 5-year survival rates ranged from 11% to 48% [6, 15, 24, 28, 46–53]. In this study, initial metastasis was a univariate prognostic factor for OSR and MFSR for soft tissue PRS as observed in the previous literature [54]. Prognosis was reported to be associated with site of the lesion [5, 55], and the immunocompromising effect and altered regional environment after the previous treatment for primary disease [23]. PRS most commonly arises at unfavorable sites, such as central trunk, or pelvic and shoulder girdles [5, 15, 26, 39]; therefore, the diagnosis is delayed [23, 39], and curative resection might be difficult to achieve [5, 15]. In this study, a wide surgical margin was almost found to be a prognostic factor for bone PRS as it was a significant factor in previous researches [54, 56]. Moreover, circumjacent abundant vascularity and lymphatics in the girdle region might facilitate metastasis and result in worse prognosis compared to the extremity lesions [57]. Immunosuppression, lymphatic obstruction, vascular insufficiency, or fibrosis could also shelter the malignant cells from the immune mechanism [23], which may cause the secondary lesions to be poorly differentiated and aggressive [39]. Other prognostic factors were also reported in previous literatures [46, 49, 54, 56]. Older age was reported as a prognostic factor for poor survival [56] and in this study it was almost found to be a univariate predictor of MFSR and OSR in bone PRS. While a previous report demonstrated that survival difference between bone and soft tissue PRSs was not significant [49], another recent report showed that the 5-year survival rates were 52% for bone PRS and 15% for soft tissue PRS, and the difference was significant in univariate analysis [28]. On the contrary, the OSR and MFSR in patients with soft tissue PRS was even higher in this study. Lager lesion was also proposed to be a survival predictor in previous reports [46, 54] and it was almost analyzed to be a univariate prognostic factor for OSR in patients with the bone PRS in this study. Although most of the patients who had not undergone surgery in this study did not have initial metastasis, the median age was high, the tumors were bone sarcomas located in difficult-to-access areas such as the pelvis and skull, and the median lesion size was large. In addition, histologic type and grade and symptom duration were suggested to be associated with prognosis [46, 56].

Although PRS management should be discussed in a multi-disciplinary setting, achieving wide surgical margins may be a unique clinical goal in most cases. PRSs are radio-resistant [23, 39, 46, 49], and the tissues surrounding these tumors have already been irradiated. Fibrosis due to irradiation also diminishes the chemotherapy effect [13, 23, 24, 39, 50, 51, 56, 58]. Because a considerable proportion of patients are elderly, the balance between potential risks and benefits of systemic therapy should also be reviewed [15]. Previous studies have shown that chemotherapy alone was only a palliative solution and it had no impact on prognosis [12, 13, 15, 23, 24, 39, 49–51], and that postoperative chemotherapy had no association with higher survival rate [13, 23, 24, 39, 50, 51, 58]. A previous study recommended reserving complete excision of the field denatured by radiation based on the concept that recurrence would develop in deep tissues or at margins of a generous resection [15]. However, we currently believe that it would be more effective to excise as much surrounding fibrotic denatured tissues as possible around PRS for delivery of chemotherapeutic agents to the target and for eliminating the possibility of additional malignant transformation. A close follow-up is understandably required for tendency for high local recurrence [15].

Although we conducted a multi-national, multi-institutional study in which 10 tertiary centers from four Asian nations had participated, the number of cases collected was less than expected despite the strict inclusion and exclusion criteria of this study. Furthermore, as patients with malignancy such as breast cancer normally have favorable prognosis, or those currently receiving radiation treatment have improved prognosis, they survived for a long period adequate enough to experience the oncogenic effects of radiation [17, 21, 27, 37, 38, 42], with aggravation of the relative risk with increasing time [37]. It may be because of the long latency period, a clinically short follow-up period, inadequate medical records without detailed previous medical history, and lack of awareness regarding PRS resulting in failure to recognize and report cases [5]. There could be still a small number of long-term survivors, because the Asian medical environment is very heterogeneous despite well-developed health services in few countries [8]. Looking on the brighter side, recent high-precision radiotherapy, which can increase the radiation dose delivered into tumors while maximizing the protection of normal tissues as much as possible, could be a reason [59].

## Conclusions

To the best of our knowledge, this study is the largest review on PRS in an Asian population although it failed to find ethnic differences. Radiotherapists should avoid unnecessary radiation exposure. Early recognition through long-term surveillance and a high degree of awareness regarding PRS are current unique keys to optimal and timely management by a multidisciplinary team. Clinicians should be concerned about this entity and they should make efforts to understand the real status of PRS.

## Supporting information

S1 Table Patient details. (XLS)
